# Prostaglandin F_2α_ stimulates the growth of human intermediate hair follicles in *ex vivo* organ culture with potential clinical relevance

**DOI:** 10.3389/fphys.2025.1556431

**Published:** 2025-06-18

**Authors:** Ben H. Miranda, Karzan G. Khidhir, Desmond J. Tobin

**Affiliations:** ^1^ Plastic Surgery & Burns Research Unit, Centre for Skin Sciences, University of Bradford, Bradford, United Kingdom; ^2^ St Andrew’s Anglia Ruskin (StAAR) Research Group, Anglia Ruskin University, Chelmsford, United Kingdom; ^3^ Department of Biology, College of Science, University of Sulaimani, Kurdistan Region, Iraq; ^4^ Charles Institute of Dermatology, School of Medicine, Dublin, Ireland; ^5^ Conway Institute, University College Dublin, Dublin, Ireland

**Keywords:** hair loss (alopecia), prostaglandin (PGF2a), intermediate hair follicles, balding or thinning hair, plastic surgery & cosmetic surgery, hair regeneration, hair loss treatment, dermatology

## Abstract

**Background:**

Hair plays a crucial role in social and sexual communication; hair disorders such as alopecia or hirsutism can therefore cause psychological distress. Current treatments are limited by unwanted side effects and a lack of understanding of hair follicle (HF) regulation, particularly in miniaturised intermediate or vellus-like follicles; the clinical targets in hair loss disorders. The discovery that bimatoprost, a prostamide F_2α_ analogue, stimulates eyelash growth suggest a possible role for other prostanoids in hair growth.

**Objectives:**

To evaluate the impact of the naturally occurring prostaglandin F_2α_ (PGF_2α_) on human intermediate HF growth, comparing the effects on matched terminal and intermediate follicles using a pre-clinical *ex vivo* organ culture model. Furthermore, to determine the involvement of PGF_2α_ receptors (FP) and their location within both these HF types.

**Methods:**

Matched human female pre-auricular facelift skin HFs were incubated with PGF_2α_ alone or in combination with an FP antagonist for 9 days in the gold-standard *ex vivo* HF organ culture model. To confirm FP gene expression in both terminal and intermediate lower HF bulbs, RT-PCR was performed using specific FP primers, confirmed by sequence analysis. Immunohistochemistry was conducted using frozen sections to locate the FP protein in HF components.

**Results:**

PGF_2α_ (100 nM) stimulated terminal HF fibre growth by 4.93% (p = 0.019) with a greater effect (10.03% (p < 0.001) stimulation) on intermediate HFs. PGF_2α_ stimulation significantly prolonged anagen (the growth phase of the hair cycle) duration in both HF types and to similar extent. These increases in hair fibre elongation were blocked by the receptor (FP) antagonist in both terminal and intermediate follicles. RT-PCR confirmed FP gene expression and immunohistochemistry located FP protein in the dermal papilla and connective tissue sheath of both intermediate and terminal HFs.

**Conclusion:**

We demonstrate, for the first time, that PGF_2α_ stimulates human HF growth in organ culture via a receptor-driven mechanism, probably directly affecting the follicles’ regulatory dermal papilla function. The greater response of intermediate, compared to matched terminal, HFs suggests potential future clinical significance for medical conditions such as alopecia, or insufficient beard growth, and promoting hair growth in ‘relatively hairless’ donor graft skin or transplant follicles after elective, trauma or burn injury surgical reconstruction.

## Introduction

Scalp hair plays an important role in human social and sexual communication, and disorders like alopecia or hirsutism can cause significant psychological distress and reduced quality of life (Randall, 2007; Randall, 2008; Aukerman and Jafferany, 2023). Non-pharmaceutical or non-surgical approaches for hair loss primarily involve concealing alopecic regions, e.g., comb-over hairstyles or utilising hairpieces (Beehner, 2013). Medical treatments have limited effectiveness and/or may be associated with side-effects. The three FDA- approved drugs for hypotrichosis have different origins and mechanisms (Devjani et al., 2023). Minoxidil, initially developed as a vasodilator, opens hair follicle (HF) associated potassium channels (Nusbaum et al., 2013; Shorter et al., 2008a; Randolph and Tosti, 2021), while finasteride, a 5α-reductase inhibitor evolved from benign prostatic hyperplasia treatment, reduces testosterone conversion to the more active form, 5α-dihydrotestosterone (Kaufman et al., 1998; Gupta et al., 2022). Lastly, bimatoprost, a prostamide F_2α_ analogue, used for treatment of eyelash hypotrichosis, and initially a glaucoma therapy, can increase human scalp terminal HF growth in whole organ culture via direct action on the prostamide F_2α_ receptor (Khidhir et al., 2013a; Subedi et al., 2022). Furthermore, some clinical reports have indicated effects of other medical treatments, such as platelet-rich-plasma (PRP), herbal extracts or their metabolites, on promoting hair growth and reducing hair loss (Wadstein et al., 2020; Gentile et al., 2017; Dhariwala and Ravikumar, 2019). Although time-consuming, HF micrografting enables delicate reconstruction, even in previously diseased areas as seen in scalp hair transplants; this is due to the unique nature of gene expression of single HFs, in part as they retain characteristics of their originating body site location (Rose, 2015; Saxena and Savant, 2017; Miranda et al., 2010; Miranda et al., 2011). More traditional surgical techniques, e.g., using rotation flaps or tissue expanders, remain important for reconstructing large areas (Krishna et al., 2023; Jayapaul et al., 2018).

New therapeutic approaches are hampered by our still-limited understanding of hair follicle bio-physiology. Specifically, human studies have largely been restricted to the large-calibre often pigmented terminal scalp HF that is much easier to microdissect and manipulate in whole-organ culture (Shorter et al., 2008a; Philpott et al., 1990; Anastassakis, 2022a; Anastassakis, 2022b). HFs are very prone to change in response to factors such as hormonal stimulation, ultimately altering the hair fibre produced (Randall, 2007). For example, under the influence of androgen stimulation during puberty, vellus HFs that produce the short and fine hair on a child’s face develop into intermediate-sized and eventually terminal HFs, producing longer, visible but low calibre and coarse hair fibres (e.g., man’s beard) respectively (Randall, 2008; Grymowicz et al., 2020). Paradoxically in genetically-predisposed patients with androgenetic and female-pattern alopecia, terminal HFs in androgen-sensitive scalp areas gradually respond to adult androgen levels by miniaturising to transform into intermediate and eventually tiny vellus-like HFs as seen on bald scalp skin (Hamilton, 1951; Whiting et al., 1999; Batrinos, 2014). HFs accomplish these transformative morphological changes during successive hair growth cycles (Saitoh et al., 1970). This cycle involves periods of active growth (anagen), regression (catagen), relative “quiescence” (telogen) and shedding (exogen) (Oh et al., 2016). In general, as the duration of anagen shortens, so too does the hair fibre length and calibre; HFs that remain in anagen for longer producing longer thicker hair fibres (Whiting, 2001).

Hair loss treatment *in vivo* is almost entirely targeted at small vellus-like or intermediate HFs, aiming to stimulate their transformation to terminal HFs. Despite that, there has been little research focus on these non-terminal human HF subtypes (Anastassakis, 2022b). Vellus-like and other miniaturised HFs are theoretically the ideal study model for hair loss investigations, as these represent the progressing or end point of the balding process. However, vellus-like HFs are so small, that their microdissection, manipulation and growth in organ culture is impractical. Intermediate HFs however, while still being technically extremely challenging to micro-dissect and culture as whole organs, have been morphometrically characterised and offer a more realistic model system (Miranda et al., 2010). Previous human studies have explored the relationship between HF morphologies and their associated hair fibre types; terminal anagen HFs extend deep into the skin’s subcutaneous fat layer and have significantly larger bulb diameters than vellus HFs (Vogt et al., 2007). Studies examining the relative size of HF components have demonstrated correlations between hair fibre calibre and length, volume and cellularity of the HF dermal papilla and hair bulb matrix size (Elliott et al., 1999). Intermediate HFs also exhibit morphological differences to terminal HFs, penetrating to a more shallow depth in the skin dermis, exhibiting lower volume HF cellularity, and importantly retain significant, biologically-relevant differences *in vitro* in organ culture (Miranda et al., 2010). Notably, intermediate HFs at the scalp/facial interface were the first human organ to show an appropriate response to a relevant hormone (testosterone), in organ culture (Miranda et al., 2018).

The role of prostanoids in HFs is not well understood. Eyelash HFs *in vivo* and scalp terminal HFs in *ex vivo* organ culture can be stimulated to produce more hair fibre by prostamide F_2α_ (bimatoprost) (Khidhir et al., 2013a; Tauchi et al., 2010; Cohen, 2010). Scalp HFs are reported to express receptors for prostaglandin F_2α_ (PGF_2α_) and prostamide F_2α_ *in vivo* (Khidhir et al., 2013a). The aim of this study is to expand our understanding of prostanoid function in human skin by investigating whether the natural prostaglandin, PGF_2α_, affects the growth (as assessed by hair fibre elongation) of the more clinically-relevant human intermediate HF in organ culture. We also aim to determine whether such effects are mediated via PGF_2α_ receptors (FP), investigating FP receptor location, and comparing findings between matched human intermediate and terminal HFs.

## Materials and methods

### Skin samples

**FIGURE 1:**
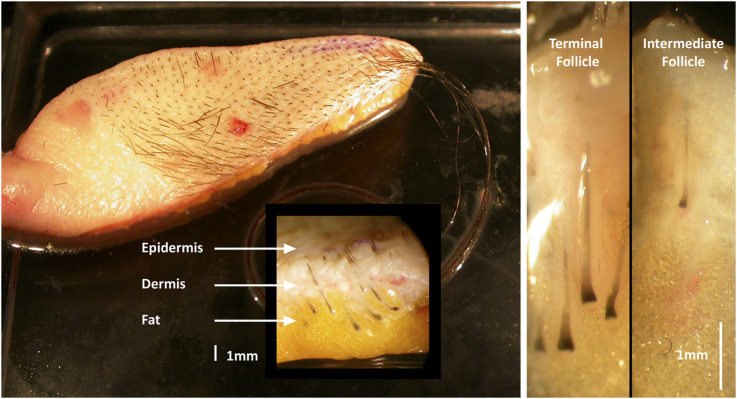
Human adult female pre-auricular facelift skin. Terminal hair follicles (HF) are larger and extend deeper into the sub-dermal fat than intermediate HFs.

Donor-matched terminal and intermediate HFs were micro-dissected from fresh human female (n = 6) pre-auricular facelift skin sourced from elective (cosmetic) plastic surgery (age range 49–65 years) (Figure 1). For organ culture investigations, samples were collected into sterile universal tubes (25 mL or 50 mL) containing basic culture medium: William’s E medium supplemented with 10μg/mL insulin, 10ng/mL hydrocortisone, 2 mM L-glutamine (Life Technologies, Paisley, UK), and 10U/mL penicillin. Unless specified, Sigma-Aldrich (Dorset, UK) supplied all materials. Skin specimens were transported on ice and stored at 4°C until HFs were isolated within 24 h of removal from the donor. For molecular biological investigations, small skin samples (about 1 cm^3^) were placed into sterile universal tubes (10 mL) containing RNA stabilization solution, RNA*later*, to inhibit RNase activity. They were kept at 4°C overnight to allow tissue penetration by RNA*later* before storage at −20°C until analysis. For immunohistochemical investigations, skin samples were similarly collected and cut into small pieces, embedded in optimal cutting temperature (OCT) compound (Raymond A. Lamb; ThermoFisher Scientific), and stored at −80°C until used.

### Microdissection of terminal and intermediate hair follicles

**FIGURE 2:**
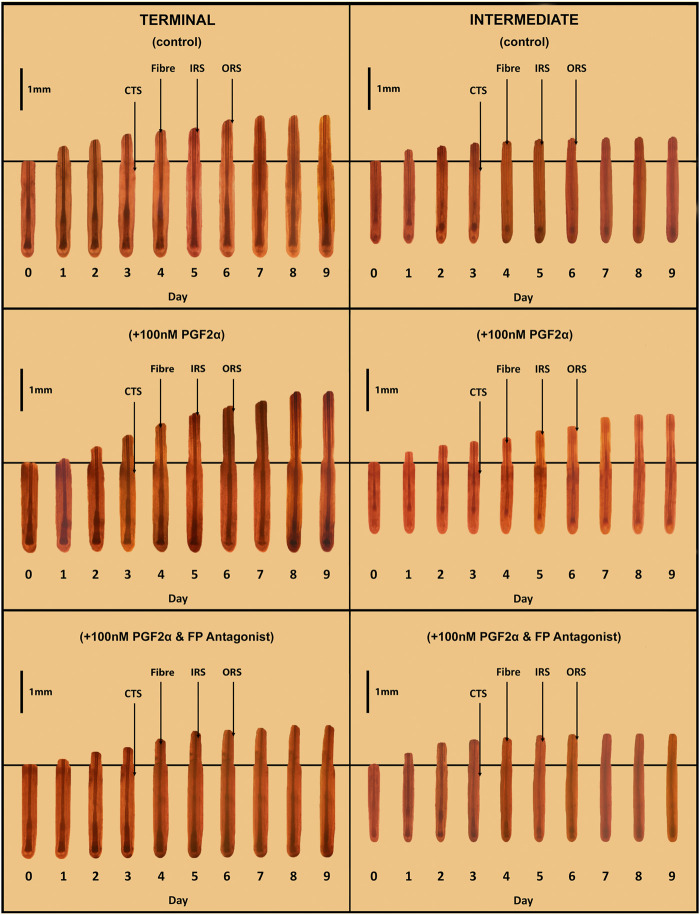
Sequential photo-micrographs of terminal and intermediate hair follicles (HF) in *ex vivo* whole organ culture under control, PGF_2α_-stimulated and PGF_2α_-inhibited conditions. Sequential photomicrographs, taken every 24h for 9 days, of representative individual terminal and intermediate HFs, demonstrate elongation/growth of the hair fibre (Fibre), inner (IRS) and outer root (ORS) sheaths. Note the connective tissue sheath (CTS) does not grow-out with the epithelial compartment of the HF, and so indicates the original length of the HF on isolation.

Matched terminal and intermediate anagen HFs (from the same skin specimen) were micro-dissected, despite significant technical challenge, from each human pre-auricular facelift sample under sterile conditions (Leica MZ8 dissecting microscope with fibreoptic cool illumination, Leica Microsystems, Wetzlar, Germany). Each HF was transferred to a Petri dish containing sterile phosphate buffered saline (PBS; Oxoid, Basingstoke, UK) for organ culture or into RNA*later* at 4°C for molecular biological studies. Isolated HFs were pooled into a fresh dish of cold PBS or RNA*later*, and individually cleared of attached dermis or subcutaneous fat material using 27.5-gauge sterile syringe needles. Care was taken to ensure that HFs remained undamaged during isolation to protect their viability as is essential for successful culture (Figure 2) (Shorter et al., 2008b). Matched terminal (n = 60) and intermediate (n = 80) HFs were micro-dissected from each human pre-auricular facelift sample to investigate gene expression.

### Hair follicle organ culture

*Ex vivo* hair follicle organ culture is the gold standard in human hair growth research. Isolated terminal and intermediate HFs were transferred to individual wells of 24-well culture plates in 1 mL of appropriate medium as follows. Basic culture medium (see above) was supplemented with either PGF_2α_ (100 nM; 900123P, Sigma-Aldrich) or PGF_2α_ (100 nM) + PGF_2α_ receptor (FP) antagonist (1μM; AL-8810, Sigma-Aldrich); both reagents were dissolved in 0.001% dimethyl sulfoxide (DMSO). Control (unstimulated) medium was supplemented only with 0.001% DMSO. Cultured HFs were maintained ‘free-floating’ at 37°C in an atmosphere of 5% CO_2_ and 95% air in a humidified incubator. Medium was refreshed every 3 days.

### Statistical assessment of hair follicle growth in culture

Terminal and intermediate HFs were microscopically-assessed for maintenance of anagen HF bulb morphology and photographed (Nikon Coolpix 4,500 digital camera, Nikon, Tokyo, Japan). Hair fibre elongation (our marker of HF ‘growth’) was measured every 24h for 9 days, using an inverted microscope (Leitz Labovert FS; Leica Microsystems). HFs exhibiting failure to grow within the first 3 days of the culture period were deemed damaged and “nonviable” and were excluded. After confirming normal distribution by using Kolmogorov Smirnov’s test, growth data were analysed using the Student’s paired t-test, and the percentage of HFs remaining in anagen over 9 days were analysed using ANOVA (SPSS, Chicago, IL, United States) (Miranda et al., 2018).

### FP mRNA expression in freshly-isolated terminal and intermediate hair follicles

Total RNA was isolated from fresh terminal and intermediate HFs (immediately after microdissection), as described previously (Shorter et al., 2008a) using a GenElute Mammalian Total RNA kit (Sigma-Aldrich, Dorset, England) or RNeasy Mini Kit (Qiagen, Hilden, Germany), and quality-checked by agarose gel electrophoresis (1.5%) before further purification to isolate poly (A^+^)RNA using a GenElute mRNA Miniprep kit (Sigma-Aldrich). RT-PCR was used to investigate FP mRNA expression. To remove any contaminating genomic DNA, mRNA samples were treated with DNase I (Invitrogen, Paisley, UK), and cDNAs were synthesised using the avian myeloblastosis virus (AMV) reverse-transcription system (Promega, Southampton, UK), as described previously (Shorter et al., 2008a). PCR amplification was performed using 10 μL of cDNA in a 50 μL reaction volume containing 0.3 μM concentrations of forward and reverse primers (Sigma-Genosys Ltd., Pamisford, UK), 200 μM concentrations of each dNTP (Promega), 5 μL of 10× reaction buffer (200 mM Tris-HCl, pH 8.4, and 500mM KCl; Invitrogen), 2.5 mM MgCl_2_ (Invitrogen), and 2.5U of recombinant *Taq*DNA polymerase (Invitrogen). Negative controls where nuclease-free water replaced cDNA were run with each PCR reaction. The primers were as follows: β-actin (GenBank NM_001101), forward 5′-ATCTGGCACCACACCTTCTACAATGAGCTGCG-3′ and reverse 5′-CTCATACTCCT-GCTTGCTGATCCACATCTGC-3′; FP, forward 5′-CGATGCCATCATCACAGAAG-3′ and reverse 5′-CTGAGCAGCTTCTCTGGCTT-3′.

After initial cDNA denaturation at 95°C (5 min), β-actin cycling conditions were 35 cycles of 95°C (1 min), annealing at 55°C (1 min), and 72°C (1 min) (Shorter et al., 2008b) and FP cycling conditions were 35 cycles of 95°C (30 ), 53°C (30 ), and 72°C (30 ). A final 10 min extension period at 72°C completed the thermocycling before cooling at 4°C (Khidhir et al., 2013b). PCR products were analysed by gel electrophoresis on a 1.5% Tris Acetate-EDTA (TAE) agarose gel (Invitrogen), as described previously (Shorter et al., 2008a).

To confirm product identity, PCR was repeated in a thermocycler with heated lid and products separated on a low-melting-point 1.5% agarose gel, excised, purified using the MinElute Gel Extraction kit (Qiagen, Crawley, UK), and sequenced by Geneblitz (Sunderland, UK). Results were compared with known published sequences using the U.S. National Center for Biotechnology Information (NCBI) Basic Local Alignment Search Tool (BLAST) program (http://www.ncbi.nlm.nih.gov/blast/bl2seq/wblast2.cgi).

### Immunohistochemistry

Immunohistochemistry was performed to confirm FP protein expression in HFs. Seven μm longitudinal cryosections were taken from terminal and intermediate HFs (Leica cryostat, CM 1800) and collected on poly-l-lysine-coated slides to increase adherence (Shorter et al., 2008b). Immunohistochemistry was performed as described previously (Shorter et al., 2008b), using a goat anti-human FP polyclonal antibody (sc-33364, Santa Cruz Biotechnology, Santa Cruz, CA, United States), at 1:75 dilution at 4°C for 18h. Antibodies were diluted in 1.5% normal mouse serum in PBS.

## Results

### Hair follicle organ culture

A total of 594 matched terminal and intermediate pre-auricular HFs were micro-dissected from six female facelift patients; 18 terminal and 15 intermediate HFs from each donor were analysed and photographed daily in each of the three organ culture conditions (Figure 2).

**FIGURE 3:**
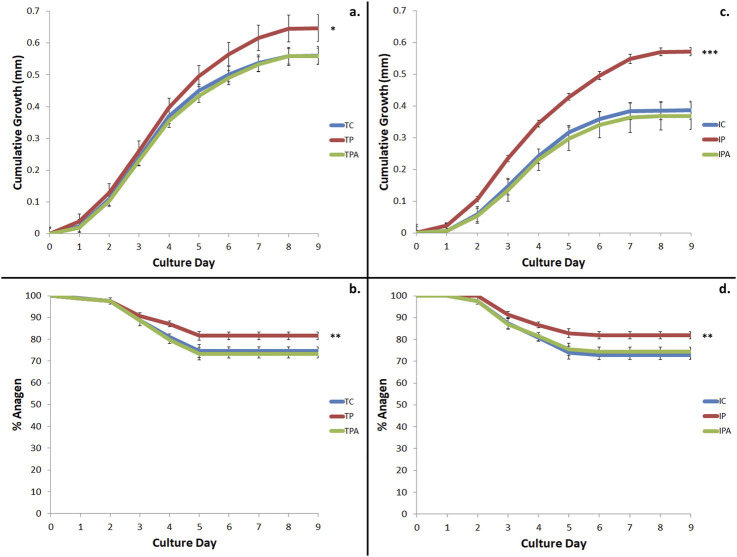
Cumulative hair growth and duration of anagen over 9 days in *ex vivo* hair follicle (HF) organ culture. **(a)** Terminal HF growth was stimulated by the addition of 100 nM PGF_2α_ (TP) versus control culture conditions (TC) (p = 0.027); this growth was inhibited by the addition of FP antagonist (TPA) (p = 0.041). **(b)** Increased terminal hair growth occurred due to a longer time HFs spent in anagen when stimulated by 100 nM PGF_2α_ (TP) versus control (TC) culture conditions (p < 0.01). This was inhibited by the addition of FP antagonist (TPA) (p < 0.01). **(c)** Intermediate HF growth was stimulated with the addition of 100 nM PGF_2α_ (IP) versus control culture conditions (IC) (p < 0.001); this stimulated growth was inhibited by the addition of FP antagonist (TPA). **(d)** Increased intermediate HF growth occurred due to a longer time spent in anagen when stimulated by 100 nM PGF_2α_ (IP) versus control (IC) culture conditions (p = 0.001); this was inhibited by the addition of FP antagonist (IPA) (p < 0.001). Of note, intermediate HFs stimulated by 100 nM PGF_2α_ (IP) grew a similar amount as hair fibre as terminal HFs under control culture conditions (TC) (p = 0.708). * = p < 0.05; ** = p < 0.01; *** = p < 0.001.

Terminal and intermediate HFs were assessed for cumulative growth and duration of maintained anagen hair bulb morphology (to indicate true hair fibre growth capacity) over the 9-day duration of the whole-organ culture assay (Figure 3). Terminal HFs produced more hair fibre than intermediate HFs under control (unstimulated) culture conditions (0.561 ± 0.006 mm vs 0.387 ± 0.029mm; p < 0.001); when stimulated with PGF_2α_, both terminal (0.647 ± 0.010mm; p = 0.027) and intermediate (0.572 ± 0.013mm; p < 0.001) HFs produced more hair fibre versus control culture conditions (Figure 3). PGF_2α_–associated stimulated growth was inhibited when terminal (0.559 ± 0.007mm; p = 0.041) and intermediate (0.369 ± 0.043mm; p < 0.001) HFs were grown in PGF_2α_ + FP antagonist conditions (Figure 3).

These above findings concurred with our observation that anagen duration was extended for both terminal (81.59% ± 1.21% vs 74.53% ± 1.92%; p < 0.01) and intermediate (81.86% ± 1.70% vs 72.82% ± 2.07%; p = 0.001) HFs when stimulated with PGF_2α_ versus control culture conditions (Figure 3). Importantly, anagen duration was subsequently reduced when terminal (73.34% ± 1.78%; p < 0.01) and intermediate (74.48% ± 1.91%; p < 0.001) HFs were grown under PGF_2α_ + FP antagonist culture conditions, versus PGF_2α_ culture conditions (Figure 3).

**FIGURE 4:**
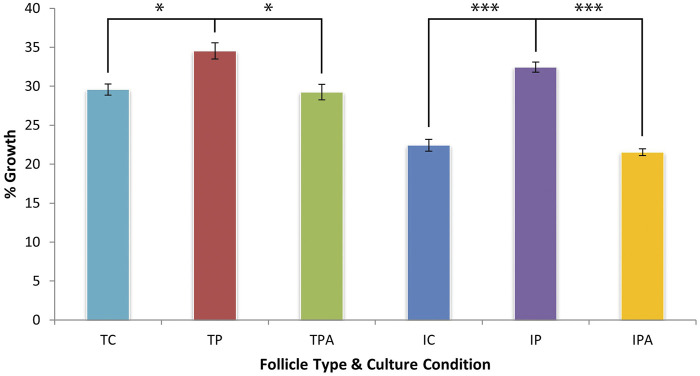
Hair fibre production over 9 days of organ culture, expressed as a percentage of initial hair fibre length. PGF_2α_ stimulated terminal HFs (TP) to increase growth significantly versus control (TC) culture conditions (p = 0.019). This stimulation by 4.93% was less compared to that of intermediate HFs; the increased growth effect was inhibited when terminal HFs were grown in PGF_2α_ + FP antagonist (TPA) culture conditions (p = 0.016). PGF_2α_ also stimulated intermediate HFs (IP) to increase growth significantly by 10.03% versus control (IC) culture conditions (p < 0.001); this increased growth effect was inhibited when intermediate HFs were grown in PGF_2α_ + FP antagonist (IPA) culture conditions (p < 0.001). Of note, intermediate HFs grown in PGF_2α_ culture (IP) conditions were stimulated to produce a similar percentage of hair fibre to terminal HFs grown in control (TC) culture conditions (p = 0.055). * = p < 0.05; *** = p < 0.001.

When hair growth was expressed as a percentage of starting HF length, terminal HFs produced more hair fibre than intermediate HFs when cultured under control culture conditions (29.57% ± 0.70% vs 22.42% ± 0.75% respectively; p < 0.001). When stimulated by PGF_2α_, both terminal (34.50% ± 1.05%; p = 0.019) and intermediate (32.45% ± 0.64%; p < 0.001) HFs exhibited greater percentage fibre growth versus control culture conditions (Figure 4). This PGF_2α_-stimulated fibre growth effect was inhibited when HFs were grown under PGF_2α_ + FP antagonist culture conditions with terminal HFs showing a 29.22% ± 0.99% (p = 0.016) change and intermediate HFs showing an even lower change of 21.53% ± 0.43% (p < 0.001). Of note, intermediate HFs grown under PGF_2α_ culture conditions exhibited a broadly similar percentage change in new hair fibre growth as terminal HFs grown in control culture conditions (32.45% ± 0.64% vs 29.57% ± 0.70%; p = 0.055) (Figure 4).

### Prostaglandin receptor expression in anagen hair follicles

**FIGURE 5:**
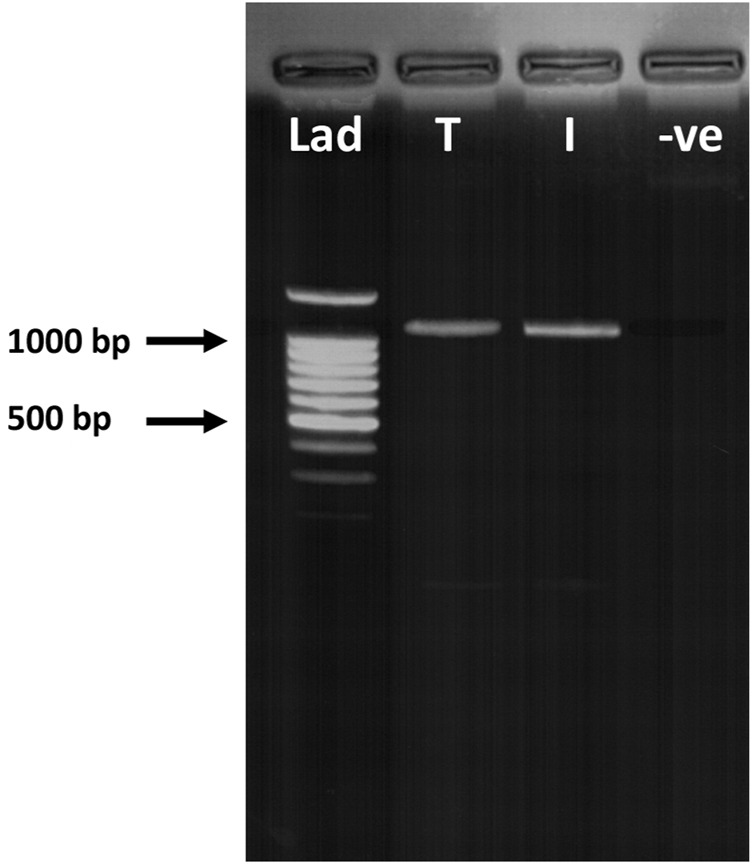
RT-PCR of terminal and intermediate hair follicles (HF). RT-PCR confirmed PGF_2α_ receptor (FP) gene expression in both terminal (T) and intermediate (I) HFs (1,080 bp). Lad = DNA ladder; -ve = negative control.

**FIGURE 6:**
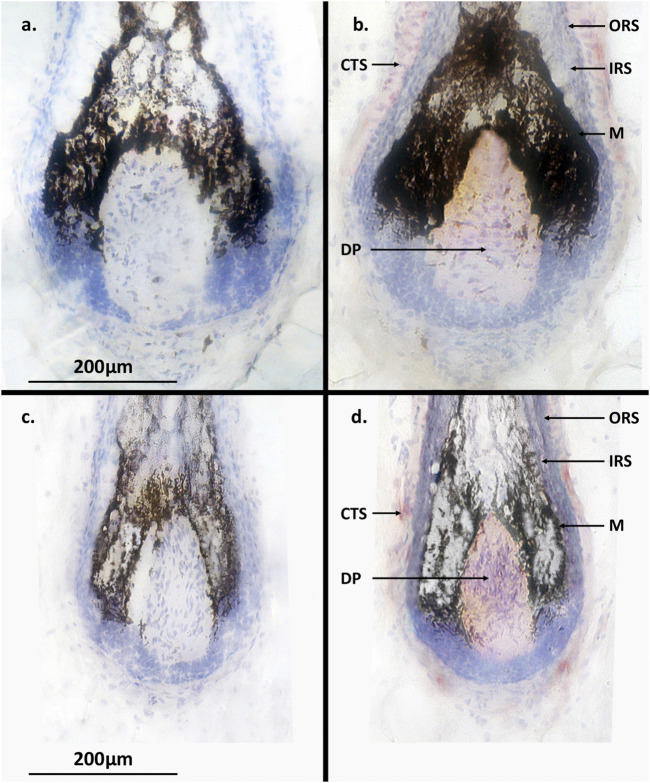
Terminal and intermediate hair follicle (HF) immunohistochemistry. **(a)** No PGF_2α_ receptor (FP) expression was detected in terminal HFs without incubation with anti-FP antibody. **(b)** Expression (pink-red staining) of FP was located in the dermal papilla and connective tissue sheath of terminal HFs but not in the epithelial cell hair matrix. **(c)** No PGF_2α_ receptor (FP) expression was detected in the control intermediate HFs. **(d)** Expression of FP (pink-red staining) was located in the dermal papilla and connective tissue sheath of intermediate HFs. ORS = outer root sheath; IRS = inner root sheath; CTS = connective tissue sheath; M = melanocytes; DP = dermal papilla.

RT-PCR and immunohistochemistry was conducted on matched terminal and intermediate pre-auricular HFs micro-dissected from six female facelift patients (Figures 5, 6). RT-PCR confirmed PGF_2α_ receptor gene expression (1,080 bp) (Figure 5), and immunohistochemical analysis located the PGF_2α_ receptor to the dermal papilla and connective sheath in both HF types (Figure 6).

## Discussion

We present the first investigation of the effects of the naturally-occurring PGF_2α_ on human intermediate HF growth in *ex vivo* organ culture. *Ex vivo* organ culture of terminal scalp HFs is the gold standard in human hair growth research and is now considered a pre-clinical assay (Miranda et al., 2010; Philpott et al., 1990; Miranda et al., 2018). The development of human HF *ex vivo* organ culture techniques was a very significant milestone in cutaneous biology research (Philpott et al., 1990). Importantly, these cultured HFs, despite being isolated from their circulation and innervation, retain for several days (typically around 7 days) their capacity to produce hair fiber at approximately *in vivo* rates. The smaller, finer intermediate HFs are relatively much more difficult to isolate and grow undamaged compared to their larger terminal scalp HF counterparts. This was evidenced by our observation that as many as one in four isolated intermediate HFs failed to exhibit hair fibre elongation during the first few days after microdissection, despite having anagen hair bulb morphology.

We demonstrate the capacity of intermediate HFs, isolated from adult human female pre-auricular skin, to respond to PGF_2α_ stimulation with increased hair growth versus their behaviour under control culture conditions; however, the degree of this stimulated growth was similar to that observed with unstimulated terminal HFs grown under control culture conditions. This provides support to the concept that intermediate HF growth is less compared to terminal HF growth, however may be stimulated to a similar rate. Moreover, antagonising FP receptors in both HF types (adding PGF_2α_ receptor antagonist) reduced PGF_2α_-associated growth stimulation to control (unstimulated) levels. Thus, it was evident that the enhanced growth effect was primarily the result of PGF_2α_ stimulation.

FP gene expression in the lower hair follicle bulb and FP protein expression in the dermal papilla and connective tissue sheath were confirmed by RT-PCR and immunohistochemistry, respectively. In that context, our findings support the view that this *ex vivo* stimulation was mediated via FP receptors located in the HF mesenchyme, particularly those in the hair-growth regulatory centre, the dermal papilla, but also in the lower connective tissue sheath (also known as the HF dermal sheath) in both terminal and intermediate HFs.

The implication of FP receptor-mediated mechanism(s) *in vivo* remain unclear, as other paracrine signalling scenarios may also occur *in vivo* (Chen et al., 2020; Kim et al., 2019; Lü et al., 2006; Bassino et al., 2015). Still, our findings align with previous studies that identified the expression of FP receptors and receptors for other stimulatory hormones within the nuclei of dermal papilla cells in terminal HFs but not in other HF anatomical (typically epithelial) components (Randall, 2007; Ami et al., 1995). The expression of FP receptors in connective tissue sheath cells aligns with our previous research (Khidhir et al., 2013a) and with the reported role of these HF mesenchymal cells in initiating new HF development (McElwee et al., 2003; Wang et al., 2014).

The dermal papilla is regarded as the organising centre of the HF, regulating HF growth and likely also pigmentation (Lei et al., 2017; Ng et al., 2022; Kwack et al., 2019). Other studies also localised key prostanoid receptors in the HF, especially within the dermal papilla, including FP, prostaglandin E_2_ receptors (EP_2_, EP_3_, EP_4_), prostaglandin D_2_ receptor (DP_2_), prostanoid thromboxane A_2_ receptor (TP), and prostaglandin I_2_ receptor (IP) (Khidhir et al., 2013a; Colombe et al., 2008; Xu and Chen, 2018). Furthermore, several studies have reported a role for prostaglandins in modulating hair growth (Johnstone and Albert, 2002; Colombe et al., 2007; Shin, 2022; Chovarda et al., 2021). For example, PGF_2α_ exhibits hair growth-promoting properties, while reduced PGE_2_ levels have been observed in androgenetic alopecia scalp tissue (Shin, 2022; Chovarda et al., 2021; Choi et al., 2015). Conversely, the prostaglandin PGD_2_ exerts hair growth-inhibitory effects and is found in elevated levels in androgenetic alopecia scalp tissue (Garza et al., 2012; Nieves and Garza, 2014). Meanwhile, bimatoprost, a synthetic structural analogue of PGF_2α_-ethanolamide, can stimulate growth (length and caliber) and pigmentation of terminal eyebrow and eyelash HFs (Cohen, 2010; Beer et al., 2013; Suwanchatchai et al., 2012; Carruthers et al., 2016; Fagien, 2010). This molecule is used widely for treating glaucoma and eyelash hypotrichosis (Khidhir et al., 2013a; Woodward et al., 2013; Wall et al., 2022; Law, 2010; Schweiger et al., 2012). Similarly, latanoprost, another PGF_2α_ analogue, is reported to induce the anagen (growth) phase and to some limited extent augment hair density in scalps affected by androgenetic alopecia (Johnstone and Albert, 2002; Choi et al., 2015; Blume-Peytavi et al., 2012; Rafati et al., 2022).

Adult female facial intermediate HFs represent an innovative, hormone-responsive human organ model that can be cultured and manipulated in the standard laboratory setting. While these intermediate HFs hold promise for fundamental scientific research investigations and drug testing, there are inherent challenges associated with their sourcing and in-lab manipulation due to their diminutive size. The relatively enormous rodent vibrissae HF has frequently been the preferred model to study developmental processes and stem cell niches (Jahoda et al., 1984; Whitehouse et al., 2002). This is because elaboration of the lower HF during the anagen phase of the hair growth cycle recapitulates many of the same molecular processes that underpin hair follicle morphogenesis including tissue regeneration from epithelial and melanocyte stem cells, under the guiding influence of the dermal papilla (Deng et al., 2021; Zhang et al., 2021; Fu et al., 2021). Still, research utilising human intermediate HFs may have significant relevance in understanding mesenchymal-epithelial biological systems in general, including cell signalling, development, stem cell manipulation, and tissue engineering. We emphasise here the importance of leveraging human HF models for hair growth studies like these. Rodent models by contrast, are highly limiting, not only because of their HFs’ miniature size, but also because these mammals do not exhibit the common hair loss disorders affecting humans (e.g., androgenetic alopecia, frontal fibrosing alopecia, etc.). Similarly, implanting individual human HFs into the skin of rodent hosts for assessment of drug effects is fraught with several confounding issues. The attempt to integrate the relatively ‘enormous’ human scalp HF into fragile and thin mouse epidermis is likely to be highly disruptive, artefactual and importantly inconsistent with retaining the human HFs in the anagen (i.e., growth) state.

In summary, our findings offer the first evidence for the impact of the natural prostaglandin, PGF_2α_, on human intermediate hair follicle growth using an *ex vivo* whole organ culture model; this seemingly occurs via action on the PGF_2α_ receptor FP via direct modulation of dermal papilla function. The potential clinical significance of these findings relates to their potential use in dermatological conditions such as alopecia and insufficient beard growth. It is unknown whether prolonged use of PGF2α may lead to tolerance or other changes to the hair growth pattern, but experience with the FDA-approved and highly-effective prostaglandin-related bimatoprost, for hair growth of the eyelashes, provides a helpful context for likely continued benefit. Furthermore, extended application to surgical reconstruction may also be beneficial, for example, after trauma or burn injury, to promote hair growth in relatively ‘hairless’ donor graft skin or transplant follicles.

## Data Availability

The raw data supporting the conclusions of this article will be made available by the authors, without undue reservation.
